# *Ribes khorasanicum*: A Potent Antioxidant Against Organ Toxicity by Effect on the NF‐κB Pathway

**DOI:** 10.1002/fsn3.4586

**Published:** 2024-11-11

**Authors:** Mohammad Naser Shafei, Sadegh Shabab, Nazanin Entezari Heravi, Reza Mohebbati

**Affiliations:** ^1^ Department of Physiology, Faculty of Medicine Mashhad University of Medical Sciences Mashhad Iran; ^2^ Applied Biomedical Research Center Mashhad University of Medical Sciences Mashhad Iran; ^3^ Department of Physiology, Faculty of Medicine Gonabad University of Medical Sciences Gonabad Iran

**Keywords:** acetaminophen, fraction, NF‐kappa B, *Ribes khorasanicum*, toxicity

## Abstract

Acetaminophen (APAP) is a well‐known drug that, in high doses, induces hepatotoxicity and nephrotoxicity. This study has investigated the preventive effect of the extract and fractions of *Ribes khorasanicum* on APAP‐induced liver and kidney damage. In this experiment, after analysis of the extract using FTIR, toxicity was induced by APAP on the 7th day. Before that, the extract and their aqueous, ethyl acetate, and n‐hexane fractions were administrated orally. 24 h after APAP administration, the animals were sacrificed. The liver and kidney were removed for the evaluation of oxidation and biochemical markers, including SGOT, SGPT, ALP, LDH, albumin, urea, creatinine, and bilirubin and also for histopathological evaluations. The safety of the extract was evaluated based on the MTT. Also, expression of the NF‐kB is done. Administration of *Ribes khorasanicum* significantly decreased the biochemical parameters compared to the APAP. Oxidative parameters, as well as histopathological changes in preventive groups, were improved compared to the APAP in both tissues. The results showed that the aqueous and ethyl acetate fractions of the extract had a better effect than the whole extract and n‐hexane fraction against APAP toxicity.

## Graphical abstract

The *Ribes khorasanicum* is a safe extract based on the MTT assay. The extract possesses various antioxidant compounds such as polyphenols and flavonoids based on the FTIR. The aqueous and then ethyl acetate fractions of the *R. khorasanicum* showed a potent antioxidant effect. Biochemical and histological changes improved with extract and fraction administration. The Nf‐Kb pathway has an important role in the prevention of toxicity.

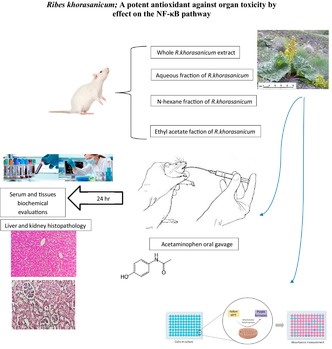

## 1 Introduction

One of the known drugs with analgesic and antipyretic effects is acetaminophen or paracetamol (N‐acetyl‐para‐aminophenol; APAP), which is being used all over the world today (Ruepp et al. 2002). APAP can be metabolized in the liver in various ways; the main ways are sulfation and glucuronidation (Dahlin et al. 1984). A small amount of it is also metabolized by cytochromes CYP2E1, CYP1A2, and CYP3A4 (Fisher and Curry 2019). The result of it is the production of N‐acetyl‐p‐benzoquinone imine (NAPQI). The NAPQI is a kind of reactive metabolite that has the potential to alter the biological function of liver cells and therefore causes toxicity (Wang, Zhang, et al. 2021a). According to studies performed on the follow‐up overdose, signs of severe centrilobular hepatic necrosis and tubular necrosis in the kidney have been seen in humans and experimental animal models (Vliegenthart et al. 2015). Under this situation, experiments have shown an increase in the production of large amounts of NAPQI, which are created by APAP metabolization via cytochrome P450s, leading to GSH depletion through NAPQI covalent binding to proteins (Qiu, Benet, and Burlingame 1998). Following these changes, an overexpressing glutathione synthetase is seen, which can lead to a hepatic GSH buildup and, finally, cause more severe hepatotoxicity and nephrotoxicity caused by APAP (Hart et al. 1995). Other experiments show that in addition to the role of protein‐binding, there are reactive oxygen species (ROS) as another important parameter that has a central role in the toxicity of APAP overdose (Wang, Zhao, et al. 2021b). As well, ROS causes increasing transcription inflammatory factors like AP‐1, IFN‐γ, and NF‐κB (Peng Zhang et al. 2020). APAP overdose can also induce vital protein dysfunction that has an important role in energy production in cells and as a result in their survival, like ATP synthetase α‐subunit, resulting in acute injury to the liver and finally either cell necrosis or cell apoptosis (Zhang et al. 2021). Although renal failure is not seen as severe as liver cell damage due to an overdose of APAP, the kidneys can also metabolize APAP through the microsomal cytochrome P‐450 system through aryl, which is smaller than liver cells (Vrbova et al. 2016). This can be confirmed by identifying additional compounds in the kidneys that are caused by the oxidation of acetaminophen to the NAPQI protein (Hart et al. 1991). In the cortex, most of the activity of the cytochrome P‐450 system can be observed, which, according to previous experiments in maximal tubes, reaches its maximum. Prostaglandin synthetase and N‐deacetylase can also be involved in renal toxicity, which is more common in necrosis of the proximal tube (Wu et al. 2009).

In this regard, the use of antioxidant and anti‐inflammatory agents can effectively improve the hepatotoxicity and nephrotoxicity induced by APAP (Ao 2020). *Ribes khorasanicum* Saghafi & Assadi (*R. khorasanicum*) (Angiosperms → Grossulariaceae → Ribes) (Hamounpeima et al. 2020) is endemic and native to the Khorasan Razavi province of Iran (Mohebbati, Kamkar‐Del, Hamounpeyma, et al. 2021; Mohebbati, Kamkar‐Del, and Shafei 2021). This plant contains many effective components, including phenols, flavonoids, and anthocyanins. It is one of the best active ingredients that is used for research and has great effects on the antioxidant system and tissue and systemic inflammation (Gholamnezhad et al. 2021). Anthocyanins have many effects, including broad antioxidant and other protective effects (Yong and Liu 2020).

In the present study, we investigate the preventive effect of the hydroalcoholic extract of *R. khorasanicum* and its fractions such as aqueous fractions, ethyl acetate, and n‐hexane extract on the acetaminophen‐induced liver and kidney damage in rats.

## 2 Methods

### 2.1 Extraction and Analysis

*Ribes khorasanicum* fruit was collected in Dargaz township in northeast Iran and was identified (Voucher number: 3242) by botanists from the Herbarium of Ferdowsi University of Mashhad (FUMH, Iran. Khorassan. Mashhad). Dried fruit was powdered and extracted using an extractor with ethanol (70%). The hydroalcoholic extract was prepared by adding 100 g of dried powder of *R. khorasanicum* to 1800 mL of ethanol 70% (540 mL distilled water and 1260 mL ethanol) using the macerating method for 72 h at a temperature of 25°C with alternated shaking. The solvent was removed under decreased pressure and incubation at 45°C temperature for 48 h (Hamounpeima et al. 2020).

Ten grams of the aqueous‐alcoholic extract of the plant, prepared by wetting, was mixed with ethanol to form a highly polar compound until uniform, then the resulting solution was transferred to a separating funnel. The solution in the separating funnel was extracted once with ‐N hexane (each time 1/3 of the volume in the separating funnel) to separate its completely non‐polar components. The prepared fraction was placed in a water bath to remove the solvent and completely concentrate. The resulting fraction is called the N‐hexane fraction. The remaining solution from the previous step was extracted three times with ethyl acetate (each time 1/3 of the volume in the separating funnel) to separate the completely non‐polar components. The prepared fraction was placed in a water bath to remove the solvent and completely concentrate. The resulting fraction is called the ethyl acetate fraction. The remaining solution from the previous step was placed in a water bath to remove the solvent until completely concentrated. The resulting fraction is called the aqueous fraction (Shahraki et al. 2016).

**FIGURE 1:**
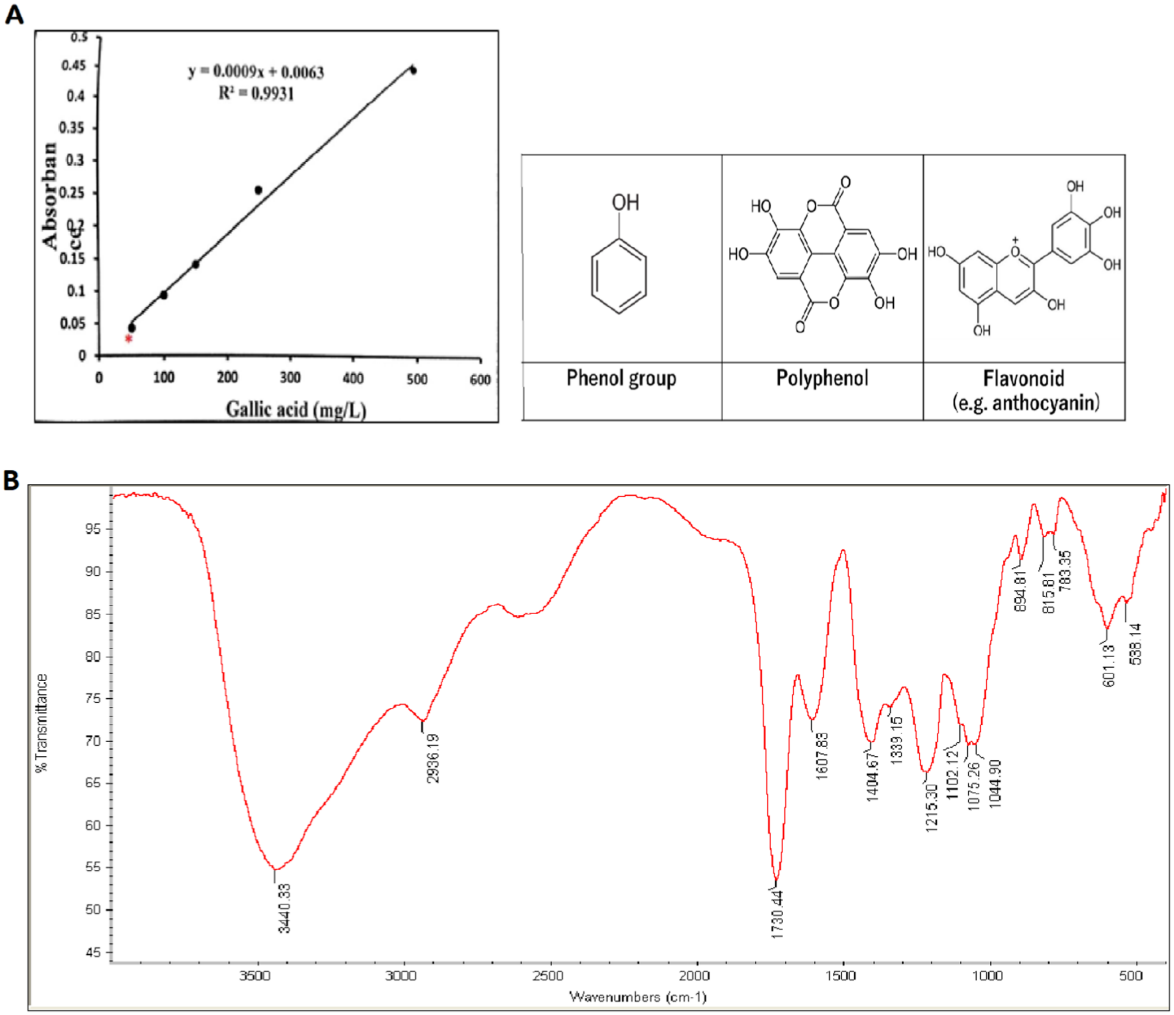
Standard curve of gallic acid. The star symbol (*) represents the amount of phenol in the extract (A). The FTIR record of the extract (B).

Based on the total phenol content, the *R. khorasanicum* extract was standardized using the Folin–Ciocalteu method (Ainsworth and Gillespie 2007). According to this method, the standard curve was provided for gallic acid, and the total phenol content was expressed as the milligram equivalent of gallic acid. The total phenol concentration in the *R. khorasanicum* extract was 42.4 mg/g crude extract (Figure 1A). The flavonoids and thiocyanin have also been assessed based on the related methods (Chang et al. 2002; Rodriguez‐Saona, Barrett, and Selivonchick 1995), and the following data were calculated, respectively: 37.9 mg/g crude extract and 9.7 mg/g crude extract.

Fourier transform infrared (FTIR) was used to identify the characteristic functional groups in the *R. khorasanicum* extract. *R. khorasanicum* extract was mixed in dry potassium bromide (KBr) and pressed at a pressure of 6 bars within 2 min to form a KBr thin disc. Then the disc was placed in a sample cup of a diffuse reflectance accessory. The IR spectrum was obtained using a Bruker Germany Vertex 70 infrared spectrometer (Figure 1B).

### 2.2 MTT Assay

The cytotoxicity of the extract on the NIH/3T3 cell line was measured by the MTT test. The MTT test is one of the colorimetric methods that is used for assessing the percentage of live cells (Pinnamaneni 2020). Briefly, NIH/3 T3 cells were seeded in a 96‐well plate (7 × 10^3^ per well), and they were allowed to attach to the bottom of the wells for 24 h. Then, different concentrations of *R. khorasanicum* extract (78.1, 156.5, 312.5, 625, 1250, 2500, and 5000 μg/mL) were prepared and added to each well for 24 h. These doses were obtained from similar studies. Afterward, MTT solution (phosphate buffer saline, 5 mg/mL) was added to each well. After 4 h of incubation, the medium was removed, and then 100 μL of dimethyl sulfoxide (DMSO) was added to each well to disintegrate the formazan precipitate. Finally, the absorption of each well was measured at 570 nm (with 620 nm as a reference) on a Stat FAX303 plate reader (Hernandez, Khandual, and López 2017). All tests were performed in triplicate. Results are represented in the IC50 value, defined as the concentration of extract that reduces 50% growth and proliferation of the cells and is used as an index in determining the toxicity effect of the extract (Fellows and O'Donovan 2007).

**FIGURE 2:**
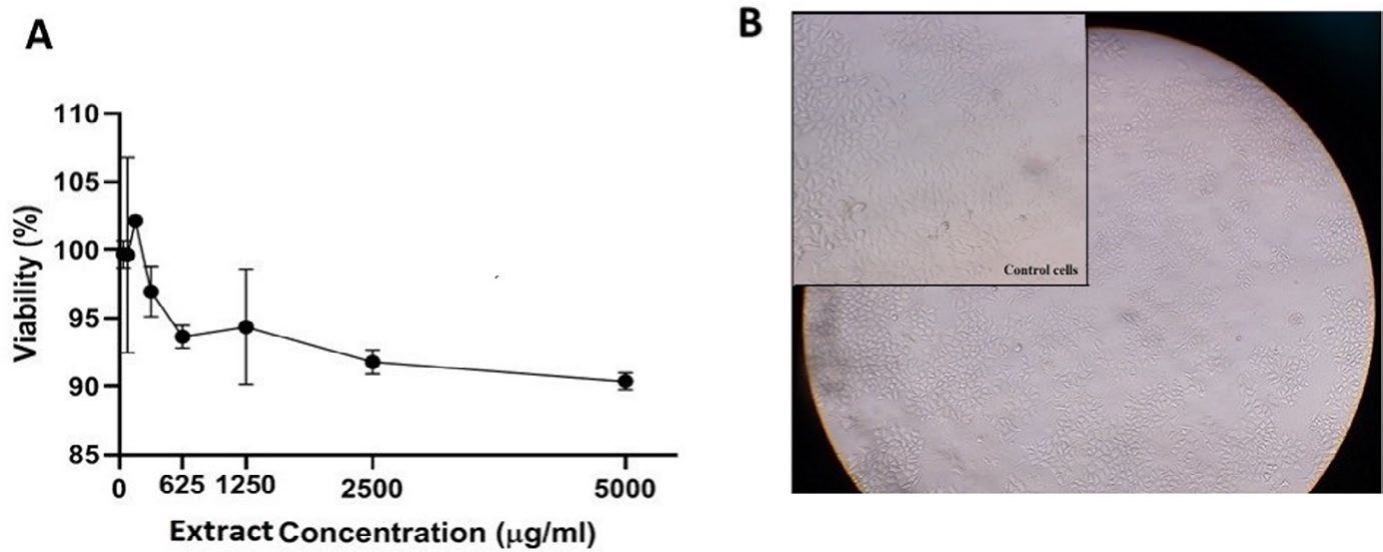
Effects of *Ribes khorasanicum* extract on cell viability of NIH/3 T3 cell line (A). Cells were treated with different concentrations of extract for 24 h. Viability was quantitated by MTT assay. Results showed the number of living cells was less reduced compared to the control wells in a dose‐dependent manner. Results are mean ± SEM. Morphological changes of NIH/3 T3 cell line after 24 h at 250 μg/mL: NIH/3 T3 cells treated with *Ribes khorasanicum* extract compared to the control (B).

In NIH/3T3 cells, 24‐h exposure to *R. khorasanicum* indicated a decrease in cell viability compared to the control group. No significant effect in cell viability was found in NIH/3T3 cells at any of these concentrations of *R. khorasanicum* compared to the control well (Figure 2).

### 2.3 Drugs and Kits

Acetaminophen was purchased from Osve Co. (Iran). Biochemical diagnostic kits including SGOT, SGPT, ALP, LDH, albumin, urea, creatinine, and direct and total bilirubin were procured from Pars Azmoon Co. (Iran).

### 2.4 Animals

Thirty male Albino Wistar rats (2–3 months) (Animal House of the School of Medicine, Mashhad University of Medical Sciences, Mashhad, Iran) weighing 250 ± 20 g at disease induction were studied. The animals were housed in a temperature‐controlled room with a 12:12‐h light–dark cycle and free access to food and water. All animal procedures were under ethical license ID: IR.MUMS.MEDICAL.REC.1400.360.

### 2.5 Experimental Protocol

The duration of the study period was 8 days to induce acute hepatotoxicity. In the prevention groups, *R. khorasanicum* extract was being received in the most effective dose (12 mg/kg) as oral gavage, and aqueous, ethyl acetate, and n‐hexane fractions of the *R. khorasanicum* were being received as oral gavage, as 20 mg/kg. In all of the prevention groups, the extract and/or fractions were administered in the first 6 days of the study (Mohebbati, Kamkar‐Del, Hamounpeyma, et al. 2021; Mohebbati, Kamkar‐Del, and Shafei 2021). APAP was administered oral gavage as a single dose of 600 mg/kg on the seventh day (Omidi et al. 2014). After 24 h, the rats were sacrificed.

Rats were divided into six groups in the following order (*n* = 5 in each group):

1. Control group: received saline on the sixth day
2. APAP group: received APAP (600 mg/kg) on the seventh day (Mohebbati et al. 2018)
3. Prevention group with *R. khorasanicum* whole extract: received whole extract (12 mg/kg) in the first 6 days and then received APAP (Extract + APAP)
4. The prevention group with an aqueous fraction of *R. khorasanicum* received this fraction (20 mg/kg) in the first 6 days and then received APAP (Aq.F + APAP)
5. Prevention group with ethyl acetate fraction of *R. khorasanicum*; received this fraction (20 mg/kg) in the first 6 days and then received APAP (EA.F + APAP)
6. The prevention group with n‐hexane fraction of *R. khorasanicum* received this fraction (20 mg/kg) in the first 6 days and then received APAP (Hex + APAP)

### 2.6 Sample Collection and Measured Parameters

On the first day, all rats were weighed, and blood samples were collected from the orbital sinus and centrifuged to obtain serum. At the end of the experimental period, the rats in all groups were weighed again, anesthetized with ether, and after withdrawing blood from the heart, a piece of liver and kidneys were removed and the animals were killed humanely. A part of the liver and one kidney were stored at −80°C to be used in future studies for oxidation marker evaluations, including total thiol (‐SH), superoxide dismutase, catalase, and malondialdehyde (MDA) (Hira et al. 2017). The other part of the liver and kidney was moved to a 10% formalin solution for pathological study. Serum samples were used by biochemists to determine the concentrations of liver and serum enzymes, including SGOT, SGPT, ALP, LDH, albumin, urea, creatinine, and direct and total bilirubin, with relevant kits provided from Pars Azmoon Co. (Iran). These kits evaluate biochemical parameters using the photometry method.

### 2.7 Western Blotting

To check the gene expression at the protein level, using the SDS‐PAGE method, the culture medium sample was electrophoresed on an acrylamide gel after the necessary steps, transferred to a PVDF membrane (Roche‐Germany), and then reviewed using specific antibodies (Santa Cruz Biotechnology). To investigate the expression of NF‐kB transcription factor at the protein level, the total cell protein was extracted so that the amount of this protein in the whole cell (both in the nucleus and in the cytoplasm) was evaluated. This factor has different p50, p65, and RelB subunits that exist as homodimer and heterodimer complexes. Considering that in this study, the measurement of the change in the expression of this transcription factor was considered, so a polyclonal antibody against the NF‐kB p65 subunit was used to identify this factor (Santa Cruz Biotechnology).

### 2.8 Oxidative Parameters Evaluation

In order to measure MDA concentration, kidney and liver tissues were weighed and immediately homogenized with potassium chloride solution. First, we mix 15 g of TCA with 2 mL of hydrochloric acid and 375 mg of TBA and make it to 100 mL with distilled water. Then, 1 mL of tissue homogenate was mixed with 2 mL of the prepared reagent and heated in a Bain‐Marie for 50 min. After cooling, the samples were centrifuged at 1000 *g* for 10 min, and their absorbance was read at a wavelength of 535 nm. Tissue MDA concentration was calculated using the formula.

Kidney and liver tissues were weighed and immediately homogenized with potassium chloride solution. Then, 1 mL of Tris‐EDTA buffer was mixed with 50 μL of tissue homogenate, and its absorbance at 412 nm wavelength was read against the blank (A1). Then, 20 μL of 10 mM DTNB reagent (in methanol) was added to it, and after 10 min at the temperature of the laboratory, the absorbance of the sample was read again (A2). The absorbance of DTNB solution was also read as a blank alone (B). DTNB reacts with SH groups and creates a yellow complex (nitromercaptobenzoate anion), which has an absorption peak at 412 nm wavelength, and its millimolar absorption coefficient is 13.6/mM/cm. The total amount of thiol groups (mM) was calculated using a formula.

Superoxide dismutase activity was measured based on the colorimetric method of Madesh and Balasurbamanian using a 96‐well plate. This method is based on the production of superoxide through autoxidation of pyrogallol and inhibition of superoxide‐dependent reduction of MTT to fromazan. The reaction is stopped by adding DMSO, which helps to dissolve the formed fromazan and stabilize the color formed.

The FRAP assay is another method for detecting antioxidant capacity. FRAP reagent was prepared using a previously described method [1,35]. In brief, 300 mM acetate buffer (pH 3.6), 10 mM TPTZ (2,4,6‐tripyridyl‐s‐triazine) solution in 40 mM hydrochloric acid, and 20 mM iron (III) chloride were mixed in a 10:1:1 ratio.

### 2.9 Histopathological Evaluations

The left lobe of the livers was embedded in paraffin, and 5 μm‐thick incisions were prepared. Each section was stained with hematoxylin and eosin (H&E), and all sections were examined by a pathologist who blinded the protocol based on inflammatory infiltration around the vessel and changes in liver fat, mild hepatic cell vacuolation, and severe necrosis (El‐Kashef and Abdelrahman 2020).

Also, the left uncapsulated kidneys were fixed in 10% formalin. It is then dehydrated and embedded in graded alcohol. 5‐μm sections were prepared in paraffin and staining was performed with H&E. Kidney sections were examined under a light microscope based on tubular necrosis and glomerular damage (Yazd et al. 2018).

### 2.10 Data Analysis

The data were expressed as mean ± SEM. Statistical analysis was done using the one‐way ANOVA followed by Tukey's post hoc test after confirmation of normality. A value of *p* < 0.05 was used to indicate statistical significance.

## 3 Results

### 3.1 Biochemical Markers Assessment

#### 3.1.1 Urea and Creatinine

**FIGURE 3:**
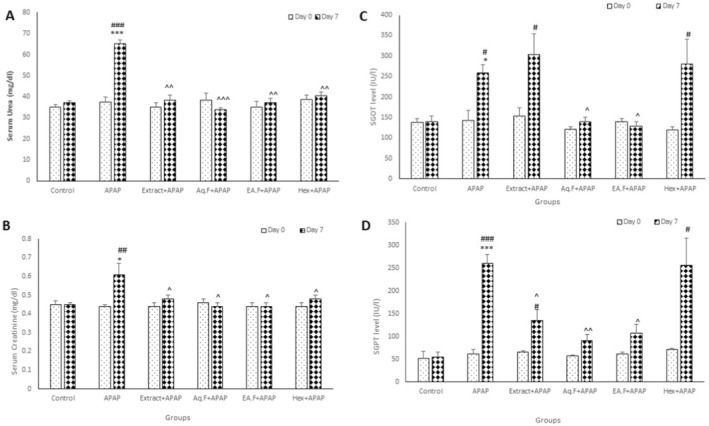
Comparison of the serum urea (A), creatinine (B), SGOT (C), and SGPT (D) in experimental groups. Data are presented as mean ± SEM (*n* = 6 in each group). *p < 0.05, **p < 0.01, ***p < 0.001 compared to the control group. ^#^ *p* < 0.05, ^##^ *p* < 0.01, ^###^ *p* < 0.001 compared to 0 day. 0. ^*p* < 0.05, ^^*p* < 0.01 and ^^^*p* < 0.001 compared to the APAP group.

Results of the current study indicated that the serum levels of urea and creatinine were significantly higher in the APAP group compared to the control group (*p* < 0.01 and *p* < 0.001). In the APAP group, serum levels of urea and creatinine significantly increased on day 7 compared to day 0 (*p* < 0.01 and *p* < 0.001). In addition, the serum levels of urea and creatinine in the Aq.F + APAP, Extract + APAP, EA.F + APAP, and Hex + APAP groups significantly decreased in comparison with the APAP group (*p* < 0.05, *p* < 0.01, and *p* < 0.001) (Figure 3A,B).

#### 3.1.2 SGOT and SGPT

The survey results showed that the serum levels of glutamic oxaloacetic (SGOT) and glutamic pyruvic transaminase (SGPT) were remarkably higher in the APAP group compared to the control group (*p* < 0.05 and *p* < 0.001). In the APAP, Extract + APAP, and Hex + APAP groups, the serum levels of SGOT and SGPT have seen a significant increase on day 7 in comparison with day 0 (*p* < 0.05, *p* < 0.01, and *p* < 0.001). And also serum levels of both of these enzymes in the Extract + APAP, Aq.F + APAP, and EA.F + APAP groups indicated a significant decrease compared to the APAP group (*p* < 0.05 and *p* < 0.01) (Figure 3C,D).

#### 3.1.3 ALP, LDH, and Bilirubin

**FIGURE 4:**
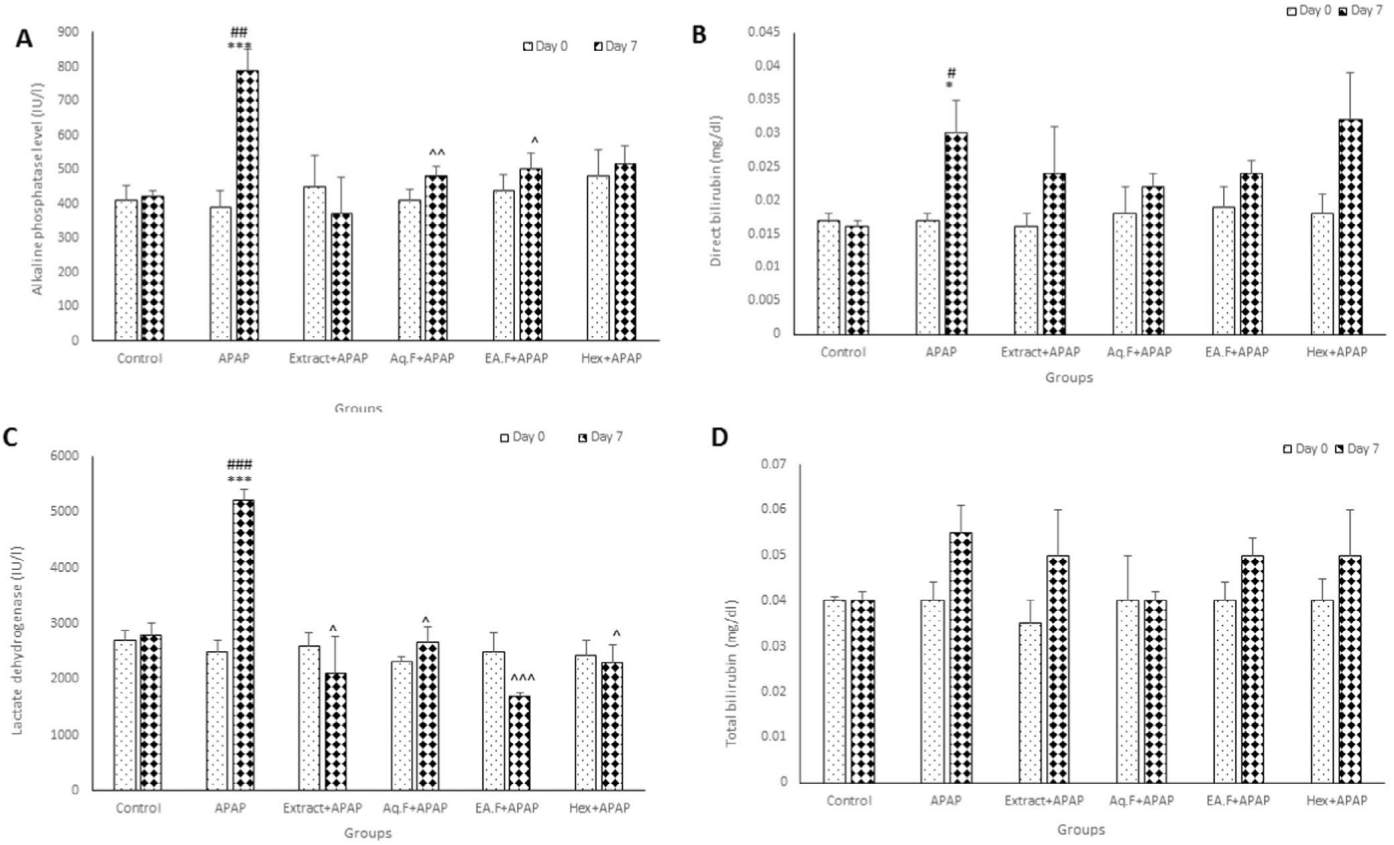
Comparison of the serum ALP (A), LDH (B), and direct and total bilirubin (C, D) in experimental groups. Data are presented as mean + SEM (*n* = 6 in each group). *p < 0.05, ***p < 0.001 compared to the control group. ^#^ *p* < 0.05, ^##^ *p* < 0.01, ^###^ *p* < 0.001 compared to 0 day. 0. ^*p* < 0.05, ^^*p* < 0.01 and ^^^*p* < 0.001 compared to the APAP group.

The results of this study also indicated that the serum levels of alkaline phosphatase (ALP) and lactate dehydrogenase (LDH) were remarkably higher in the APAP group compared to the control group (*p* < 0.001). In the APAP group, ALP and LDH significantly increased on day 7 compared to day 0 (*p* < 0.01 and *p* < 0.001). In addition, the ALP in only Aq.F + APAP and EA.F + APAP groups and the LDH in Extract + APAP, Aq.F + APAP, EA.F + APAP, and Hex + APAP had a remarkable decrease compared to the APAP group (*p* < 0.05, *p* < 0.01, and *p* < 0.001) (Figure 4A,B).

Except for the APAP group on day 7 where a significant increase in serum level of direct bilirubin (BIL D) was observed in comparison with the control group and day 0 (*p* < 0.05), there was no significant difference between the other five groups for BIL D and total bilirubin (BIL T) on different days (Figure 4C,D).

### 3.2 Oxidative Stress Assessment in the Liver and Kidney Tissues

#### 3.2.1 MDA

The results of the current study indicated that the Extract + APAP, Aq.F + APAP, EA.F + APAP, and Hex + APAP groups had a significant decrease in the liver and renal tissues for the level of MDA compared to the APAP group (*p* < 0.001). Also, there was a significant increase in the renal tissue's MDA concentration in the APAP group in comparison with the control group (*p* < 0.001), while in the liver tissue, there was no significant difference in the APAP group compared to the control group (Table 1).

**TABLE 1:** Comparison of the oxidative stress markers (MDA, tThiol, SOD, and FRRAP) in the kidney and liver tissues of experimental groups.

Oxidative stress parameters	MDA (μmol/mg)		tThiol (mmol/mg)		SOD (U/mg)		FRRAP (mmol/mg)	
Tissues	Kidney	Liver	Kidney	Liver	Kidney	Liver	Kidney	Liver
Groups								
Control	10.2 ± 0.09	16.7 ± 1.0	0.6 ± 0.03	1.42 ± 0.04	212 ± 3.1	210.3 ± 3.6	0.5 ± 0.03	0.5 ± 0.02
APAP	24.6 ± 1.1***	22.6 ± 1.3	0.09 ± 0.1**	0.11 ± 0.12***	111 ± 2.3***	120 ± 2.5***	0.2 ± 0.04***	0.1 ± 0.07***
Extract + APAP	8.8 ± 3.1^###^	3.4 ± 1.9^###^	0.3 ± 0.1	0.43 ± 0.09	376.4 ± 7.7^###^	381 ± 6.8^###^	0.5 ± 0.04^###^	0.6 ± 0.06^###^
Aq.F + APAP	6.2 ± 1.2^###^	1.4 ± 0.3^###^	0.31 ± 0.1	0.66 ± 0.1^##^	383.1 ± 4.5^###^	369.8 ± 10.3^###^	0.4 ± 0.02^#^	0.5 ± 0.03^###^
EA.F + APAP	5.8 ± 0.7^###^	4.5 ± 0.9^###^	0.18 ± 0.04	0.4 ± 0.1	379 ± 4.1^###^	359 ± 20.5^###^	0.5 ± 0.04^###^	0.5 ± 0.02^###^
Hex + APAP	6.9 ± 1.5^###^	4.3 ± 2.9^###^	0.3 ± 0.1	0.3 ± 0.01	368.7 ± 10^###^	351.8 ± 19.5^###^	0.4 ± 0.07^#^	0.3 ± 0.01^#^

*Note:* Data are presented as Mean ± SEM (*n* = 6 in each group).

**p < 0.01, ***p < 0.001 compared to the control group. ^#^ *p* < 0.05, ^##^ *p* < 0.01 and ^###^ *p* < 0.001 compared to the APAP group.

#### 3.2.2 SOD

The present results indicated a significant decrease in the liver and renal tissue's SOD activity in the APAP group compared to the control group (*p* < 0.001). In addition, the SOD activity in the Extract + APAP, Aq.F + APAP, EA.F + APAP, and Hex + APAP groups significantly increased in comparison with the APAP group (*p* < 0.001) (Table 1).

#### 3.2.3 Total Thiol

The results showed that the liver and renal tissue's level of total thiol content was significantly lower in the APAP group compared to the control group (*p* < 0.01 and *p* < 0.001). Also, it was indicated that liver tissue's level of total thiol content in the Aq.F + APAP group significantly increased compared to the APAP group (*p* < 0.01) (Table 1).

#### 3.2.4 FRAP

The results of the current study indicated that there was a significant decrease in the liver and renal tissue's level of FRAP concentration in the APAP group compared to the control group (*p* < 0.001). In addition, there was a significant increase in the FRAP concentration in the Extract + APAP, Aq.F + APAP, EA.F + APAP, and Hex + APAP groups in comparison with the APAP group (*p* < 0.05 and *p* < 0.001) (Table 1).

### 3.3 Histopathology

#### 3.3.1 Liver Evaluation

**FIGURE 5:**
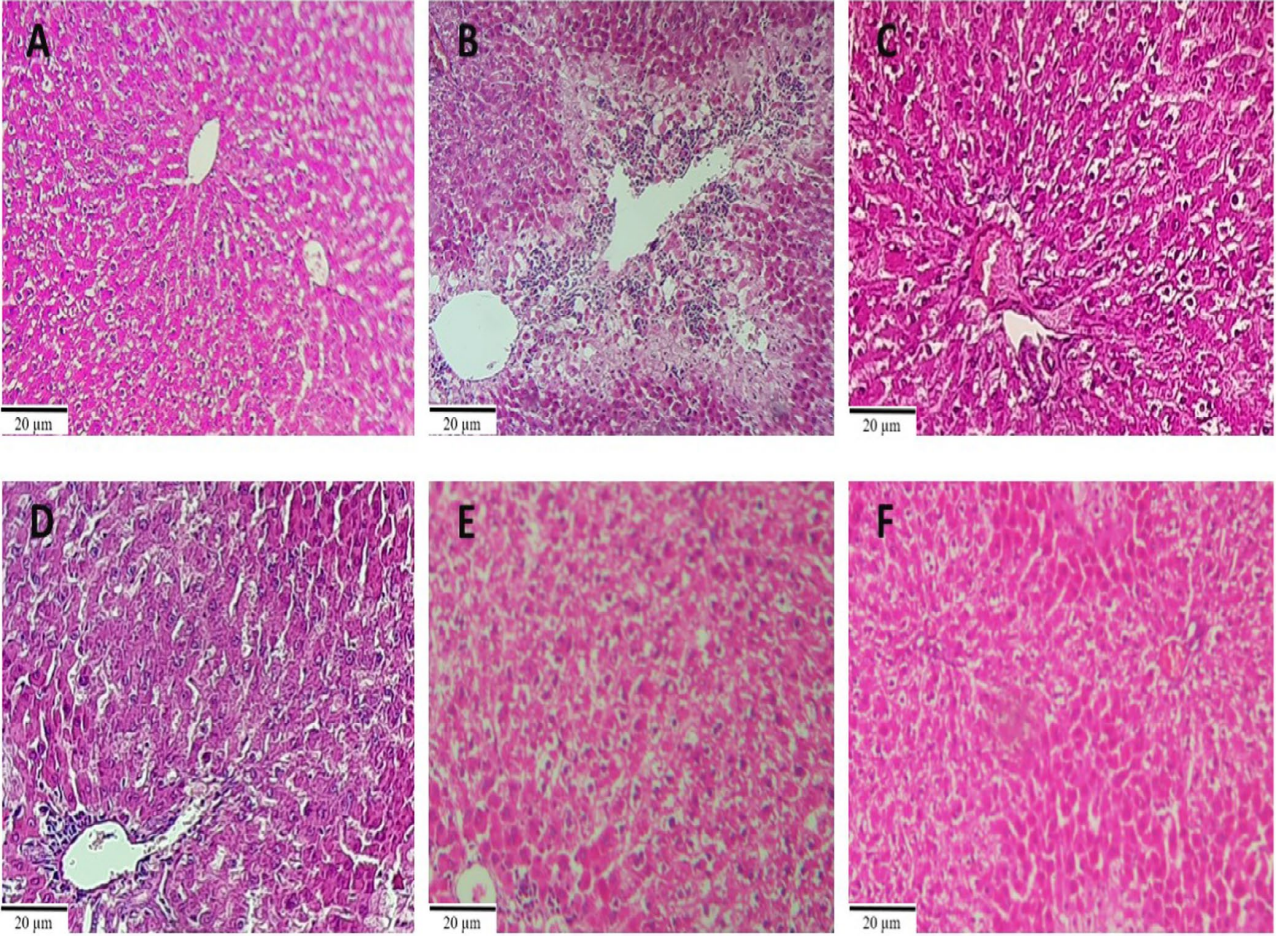
Microscopic liver sections stained with H&E. Control (A), APAP (B), Extract + APAP (C), Aq.F + APAP (D), EA.F + APAP (E), and Hex + APAP (F) groups; magnification ×40. (Scale bar = 20 μm). In image B, severe necrosis and severe vacuolar degeneration of hepatocytes were seen with an increase in Kupffer cells.

Histopathological studies showed that in the control group, liver tissue is normal and without necrosis. In the APAP group, severe tissue damage, including severe necrosis and severe vacuolar degeneration of hepatocytes, was seen with an increase in Kupffer cells. In the groups receiving the extract and its N‐hexane fraction, major lesions, including moderate hepatocyte necrosis and mild vacuolar degeneration without increasing Kupffer cells, were observed, but no major lesions were observed in the groups receiving the aqueous and ethyl acetate fractions of the extract in the liver (Figure 5).

#### 3.3.2 Kidney Evaluation

**FIGURE 6:**
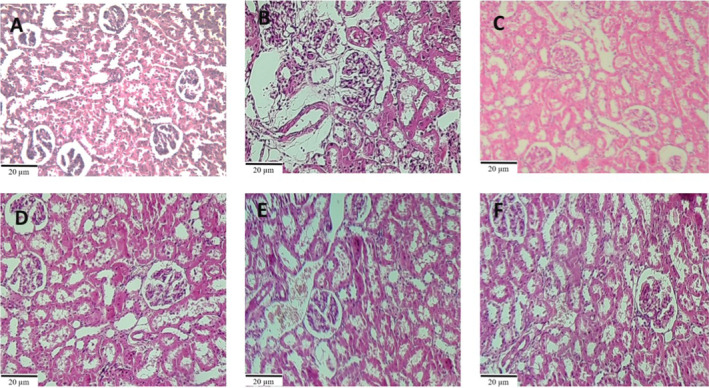
Microscopic kidney sections stained with H&E. Control (A), APAP (B), Extract + APAP (C), Aq.F + APAP (D), EA.F + APAP (E), and Hex + APAP (F) groups; magnification ×40. (Scale bar = 20 μm). In image B, severe tubular necrosis and glomerular damage are shown.

Histopathological studies about kidneys indicated that in the control group, renal tissue is normal and without lesions. In the APAP group, severe tissue damage, including severe tubular necrosis and degenerative changes, was seen with edema, congestion, and interstitial hemorrhage. In the groups receiving the extract and its N‐hexane fraction, mild tubular necrosis and degeneration were observed, and these changes in the groups receiving the aqueous and ethyl acetate fractions of the extract were minimized (Figure 6).

### 3.4 NF‐kB

**FIGURE 7:**
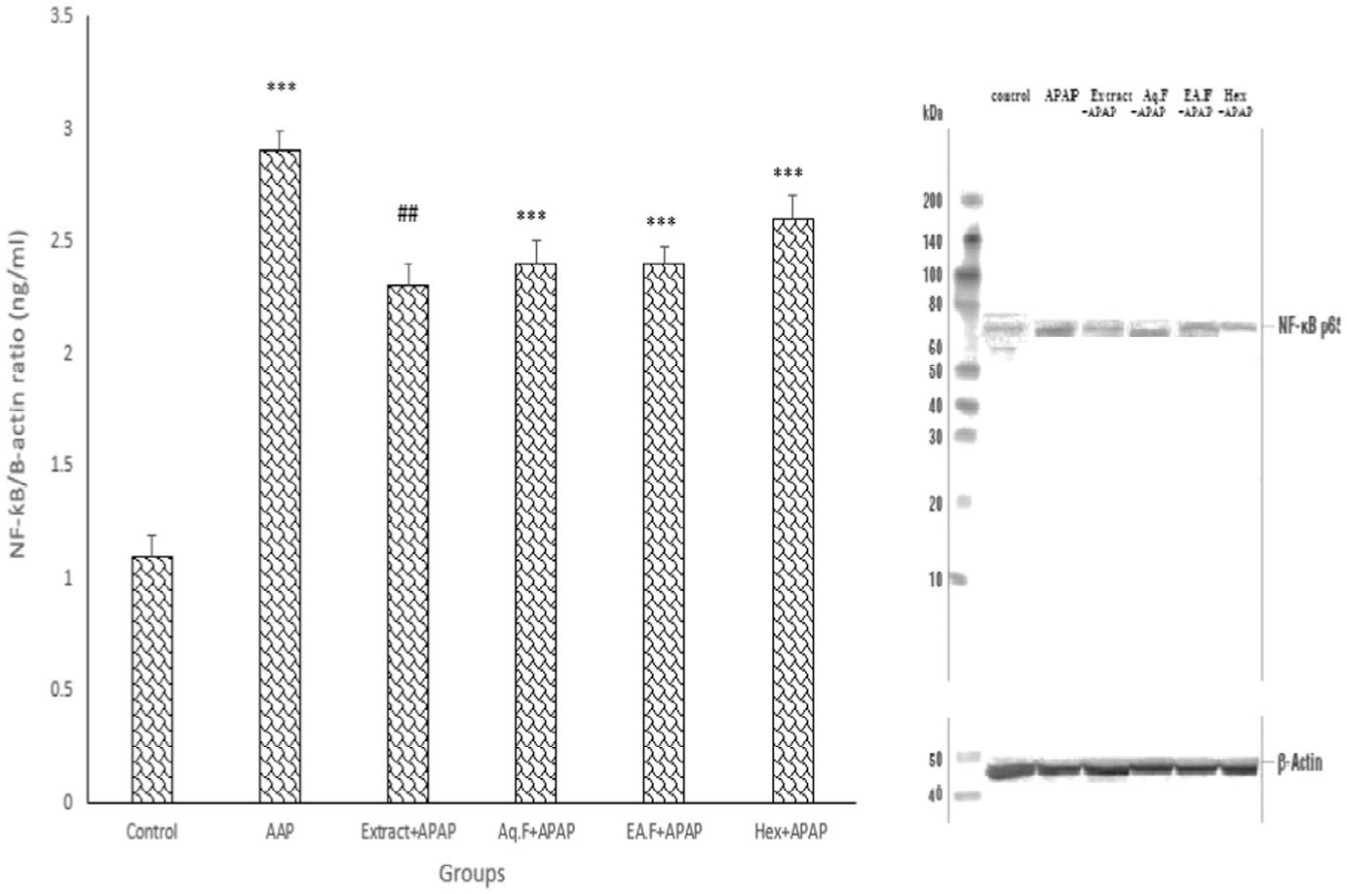
Comparison of the NF‐kB expression in experimental groups. Data are presented as mean ± SEM (*n* = 6 in each group). ***p < 0.001 compared to the control group. ##*p* < 0.01 compared to APAP.

The results indicated that this marker significantly increased in the liver tissue in the APAP group compared to the control group (*p* < 0.001). In addition, there was a significant reduction in this marker in the Extract + APAP group in comparison with the APAP group (*p* < 0.01). Also, the extract groups show a significant increase in this parameter compared to the control (*p* < 0.001) (Figure 7).

## 4 Discussion

Hepatotoxicity and nephrotoxicity are two main side effects of APAP overdose (Ellman 1959). Based on performed studies, liver absorption of the APAP leads to the over‐production of reactive oxygen species (ROS) and eventually leads to apoptosis and necrosis of the liver and renal cells (Sohail et al. 2019). Our results showed that the liver enzymes and renal parameters increased in the APAP toxicity (Akgun, Boyacioglu, and Kum 2020).

Severe hepatocyte degeneration and necrosis in the liver of APAP received rats were observed. In APAP intoxication, a great amount of NAPQI, extremely toxic to the liver, will be produced. This substance is normally detoxified via conjugation with glutathione to form cysteine and mercapturic acid conjugates (Lu et al. 2013). Under conditions of excessive NAPQI formation or hepatic glutathione depletion, NAPQI covalently binds to vital proteins and the lipid bilayer of hepatocyte membranes. However, the present discovery seems to be consistent with other research results, which found massive hepatocyte necrosis, liver failure, or death in APAP intoxication (Bunchorntavakul and Reddy 2013).

Also, the MDA level increased and the total thiol content, SOD activity, and FRAP were reduced in the liver and renal tissues of APAP‐treated animals in comparison with the control group; this change is in concurrence with previous reports (Karaali, Fahmi, and Borjac 2019). *R. khorasanicum* is known as an antioxidant and anti‐inflammatory that is a safe compound for oral administration (Hamounpeima et al. 2019). In this study, different improvement effects were seen in the evaluated markers depending on the whole extract and three types of extract fractions. The extract of this plant contains the major phytochemicals as secondary metabolites of this plant, including polyphenols as natural antioxidants and anti‐inflammatory agents and fatty acids that are different in terms of polarity (Perez‐Serradilla, Priego‐Capote, and Luque de Castro 2007). Therefore, the varying structures and polarity of these phytochemicals may affect their solubility, extraction yield, and antioxidant activities in different solvents (Ng, Samsuri, and Yong 2020). The related studies show that an aqueous fraction of the *R. khorasanicum* extract contains polar compounds, mainly total phenolic compounds. Also, the ethyl acetate and n‐hexane fractions of the *R. khorasanicum* extract contain semi‐polar and non‐polar compounds containing mainly anthocyanins and flavonoids. Thus, for further investigation of the *R. khorasanicum* effects, in addition to the whole *R. khorasanicum* extract, three fractions, including aqueous fraction (polar), ethyl acetate fraction (semi‐polar), and n‐hexane fraction (non‐polar), were also examined. Therefore, due to the presence of special solvents in all three fractions, a wide range of soluble compounds with different polarities can be found in the plant extract (Hamounpeima et al. 2019). The most important phytochemicals in *R. khorasanicum* are compounds such as flavonoids, saponins, tannins, alkaloids, anthocyanins, proteins, and ascorbic acid (Mohebbati, Kamkar‐Del, and Shafei 2021). What has been considered anti‐inflammatory and antioxidant in medicinal plants are flavonoids anthocyanins, vitamins (E and C), and reducing sugar (Yazdi et al. 2018). According to previous studies, the aqueous fraction of the *R. khorasanicum* extract contains polar compounds, mainly total phenolic compounds. The ethyl acetate and n‐hexane fractions of the extract contain semi‐polar and non‐polar compounds containing mainly anthocyanins and flavonoids (Hamounpeima et al. 2019). It is concluded that flavonoids are less polar and therefore more found in the ethyl acetate phase (Altemimi et al. 2017). It has been determined that the polar fraction gave the most total phenolic content, while semi‐ and non‐polar fractions had the lowest total phenolic content (Ng, Samsuri, and Yong 2020). Evaluations of the role of phenols as potent antioxidants in plants confirm that phenolic and polyphenolic compounds are the best free oxygen scavengers to prevent the production of free radicals (Altemimi et al. 2017). Studies have confirmed the antioxidant role of catechol derivatives of polyphenols as well as their role in the transfer of H atoms and single electrons and the chelation properties of metals of phenolic compounds (Zeb 2020). Flavonoids are polyphenolic compounds with antioxidant activity related to OH‐phenolic groups, such as epigenin, vegonin, bicalin, scotlarine, and gonoside (Saboura et al. 2014). Flavonoids reduce the production of reactive oxygen species by inhibiting pro‐oxidant enzymes such as xanthine oxidase, stimulate the nuclear factor erythroid‐derived 2 (nrf2), and a transcription factor that binds to antioxidant response elements (AREs) in the promoter region of genes encoding various antioxidants and phase II detoxifying enzymes that are known to protect against chemical carcinogenesis.

Anti‐inflammatory effects of the flavonoids have been mainly subscribed to the inhibition of the NF‐κB pathway (Owona, Abia, and Moundipa 2020). Studies showed that NF‐κB expression was inhibited by various flavonoid and polyphenolic ingredients. Based on the FTIR report, this extract has flavonoids and polyphenols that cause NF‐κB inhibition and exert anti‐inflammatory effects on tissues. The function of phenols and flavonoids as anti‐inflammatory has also been proved by inhibiting pro‐inflammatory mediators such as cytokines/chemokines, eicosanoids, and adhesion molecules (Omidi et al. 2014; Owona, Abia, and Moundipa 2020).

In a study by Zhi Xiang et al., by evaluating and also determining the power of free radical scavenging for phenols and flavonoids in different plants, it was found that the rate of scavenging of free radicals using the 2,2‐diphenyl‐1‐picrylhydrazyl (DPPH) radical scavenging method in phenols was higher than flavonoids (Zhao et al. 2008). In the current study, treatment of APAP‐administrated rats with whole *R. khorasanicum* extract (12 mg/kg) and all these fractions at (20 mg/kg) for 6 days before oral administration of APAP showed a considerable improvement in the decrease of serum levels of the urea, creatinine, SGOT, SGPT, ALP, LDH, BIL D, and BIL T in the treated groups with *R. khorasanicum* whole extract. Also, the concentration of the MDA decreased and the total thiol content, SOD activity, and FRAP increased in the liver and renal tissues of APAP‐treated animals in comparison with the control. The aqueous fraction in comparison with the other pre‐treatments has the greatest effect on the hepatotoxicity and nephrotoxicity induced by APAP. After that, to a lesser extent, the ethyl acetate fraction of the extract had an improvement effect, and the effects of the whole extract and n‐hexane were almost identical. This improvement in the parameters measured in the aqueous fraction can be attributed to polar components such as phenolic compounds, vitamin C, and soluble sugars. After that, ethyl acetate extract, due to its abundance of flavonoids, was able to make a significant contribution to improving the measurement parameters.

## 5 Conclusion

The results showed that the aqueous fraction and then the ethyl acetate fractions of the *R. khorasanicum* had a better effect on improving the hepatotoxicity and nephrotoxicity than the whole extract and n‐hexane fraction. These protective effects can be attributed to the large amounts of phenolic substances and many types of flavonoids in these two fractions.

## Author Contributions

**Reza Mohebbati:** conceptualization, methodology. **Sadegh Shabab:** visualization, investigation. **Nazanin Entezari Heravi:** data curation, writing – original draft preparation. **Mohammad Naser Shafei:** reviewing and editing.

## Ethics Statement

All animal procedures based on the arrive guideline were under ethical license ID: IR.MUMS.MEDICAL.REC.1400.360. It was approved by Mashhad University of Medical Sciences.

## Conflicts of Interest

The authors declare no conflicts of interest.

## Data Availability

The data that support the findings of this study are available from the corresponding author upon reasonable request.
